# The Geneva Brain Health Services for the prevention of dementia (dBHS): preliminary results from the pilot program

**DOI:** 10.1002/alz70860_103056

**Published:** 2025-12-23

**Authors:** Federica Ribaldi, Augusto J. Mendes, Ozge Sayin, Giovanni B. Frisoni

**Affiliations:** ^1^ Geneva Memory Center, Department of Rehabilitation and Geriatrics, Geneva University Hospitals, Geneva, Geneva, Switzerland

## Abstract

**Background:**

Over four years, the European Task Force for Brain Health Services (dBHS) has developed innovative dementia prevention services targeting cognitively unimpaired individuals at high‐risk of developing dementia, aiming to reduce their risk of cognitive impairment. The dBHS program serves as a complementary service to existing memory clinics by offering risk assessment and profiling, risk communication, risk reduction, and cognitive enhancement; the so‐called 4 pillars of dBHS. The Geneva Memory Center launched a pilot program in June 2024. This abstract presents baseline data from the first participants and their satisfaction with the initial two pillars of the dBHS.

**Method:**

Ten patients (mean age: 63years; 70%female) were invited to join the dBHS program following the standard memory clinic workup, provided they were classified as cognitively unimpaired (subjective cognitive decline or worried well). After demographic, clinical and neuropsychological data were collected in the context of the traditional memory clinic workup, scales were used to assess the 14 dementia risk factors according to Livingston et al 2024 and sleep, and blood was collected. Participants’ feedback on the risk assessment and communication sessions was collected.

**Result:**

Participants had high educational level (mean education:16years), good physical health, and high physical activity levels. The mean BMI was 23.4, with no cases of obesity, hearing loss, traumatic brain injury, diabetes, or hypertension; one participant had hypercholesterolemia. No cases of alcohol abuse or social isolation were reported; only one was a current smoker. Mood assessments indicated borderline anxiety in three participants and mild in one; borderline depression was detected in one participant. APOE genotyping in four participants revealed an e3/e3 genotype in all. The participants responded positively to the risk assessment and communication sessions, expressing clear understanding of the protocol, its purpose, and subsequent steps, as well as satisfaction and comfort with the sessions. They demonstrated interest and motivation in participating in the risk reduction program.

**Conclusion:**

The pilot cohort of the dBHS highlights the feasibility of implementing a structured, multi‐domain dementia prevention program. High participant satisfaction and adherence suggest strong potential for scaling the dBHS. Future efforts will focus on expanding the cohort, implementing risk reduction interventions, and evaluating long‐term outcomes.

